# Supplementary figures and images for: Analysis of ceRNA network of differentially expressed genes in FaDu cell line and a cisplatin-resistant line derived from it
Source: PeerJ. 2021 Jul 1;9:e11645. doi: 10.7717/peerj.11645 (PMC8255068; doi:10.7717/peerj.11645)

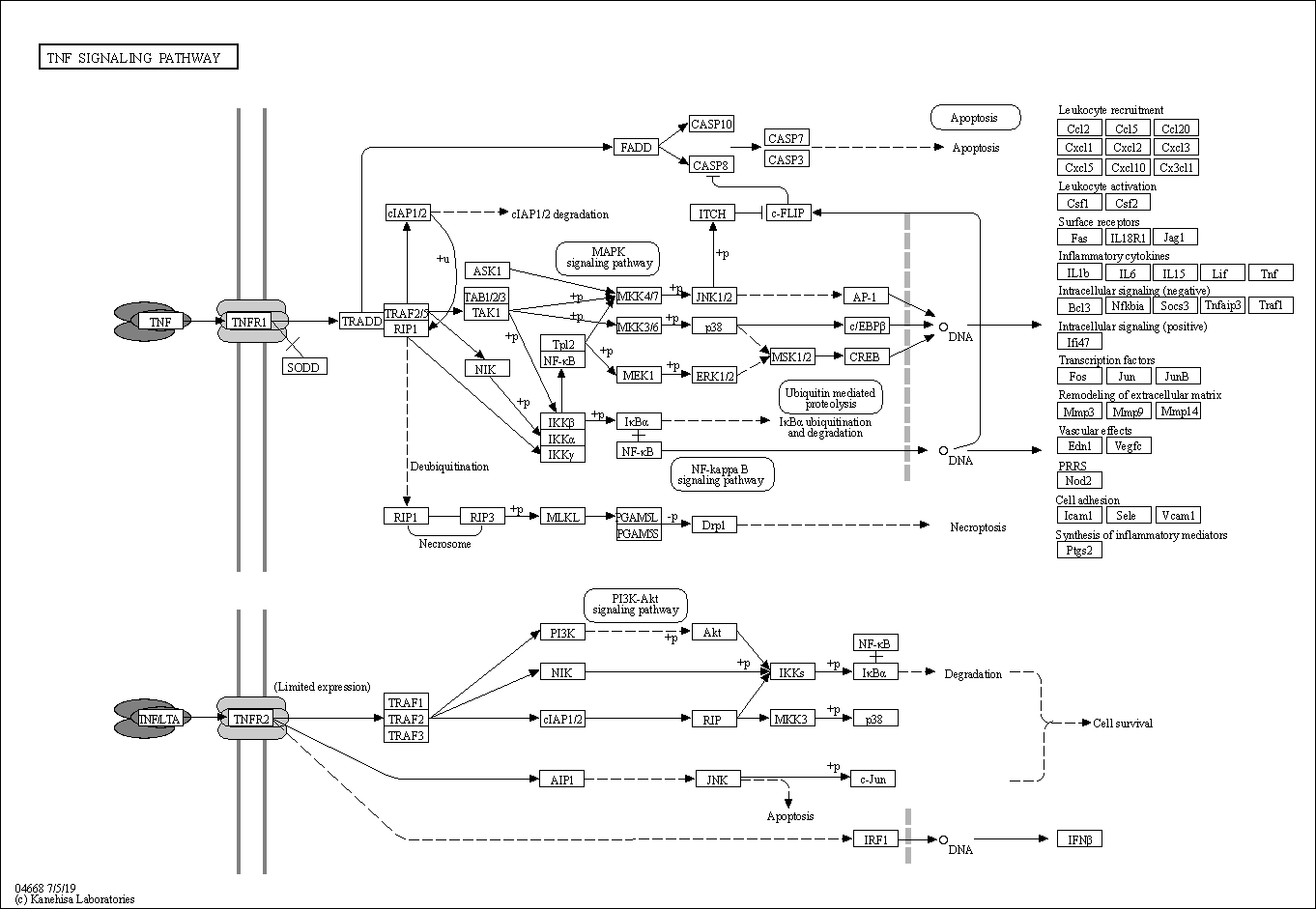

Supplement: Supplemental Information 1 [file peerj-09-11645-s001.png]

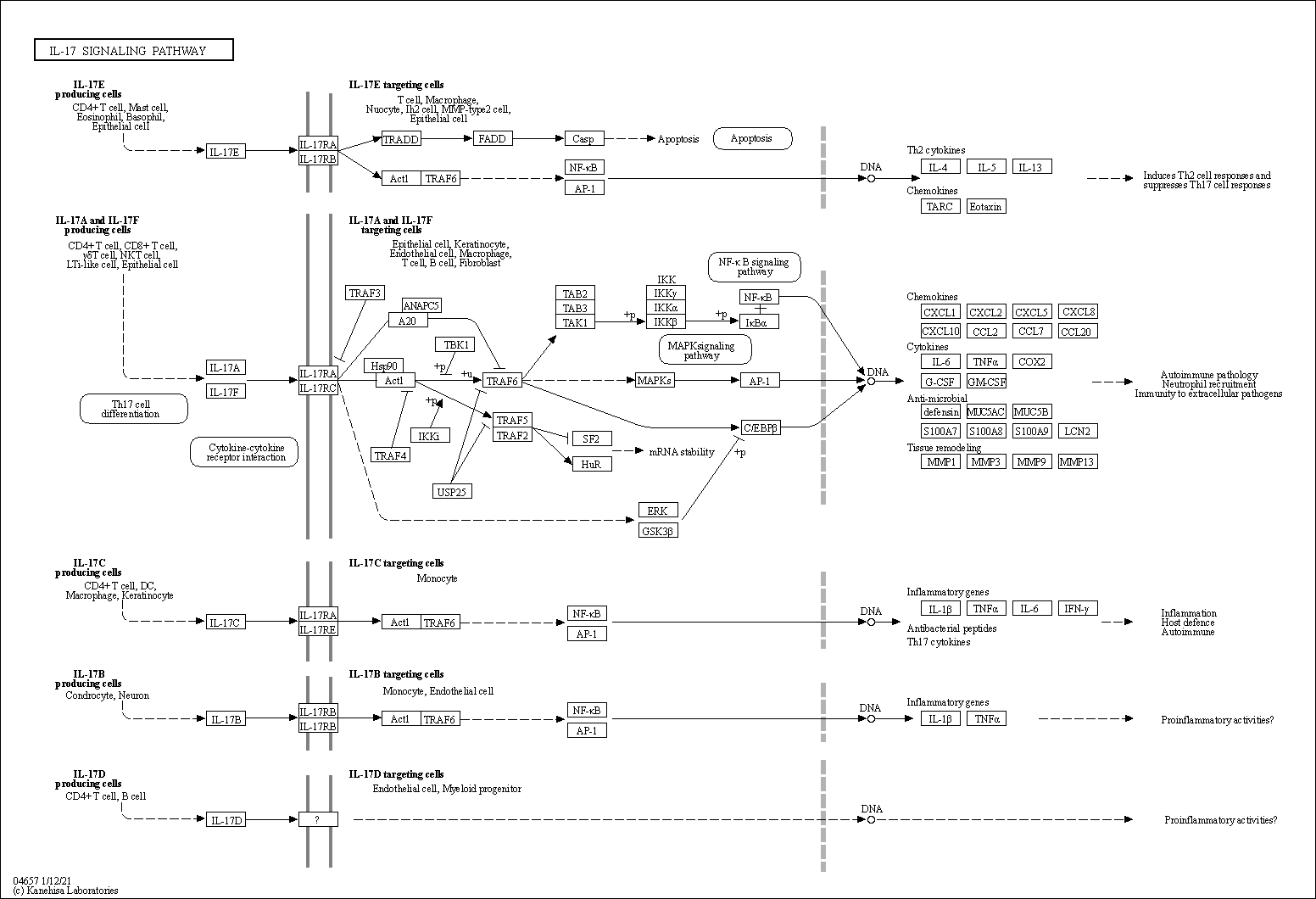

Supplement: Supplemental Information 2 [file peerj-09-11645-s002.png]

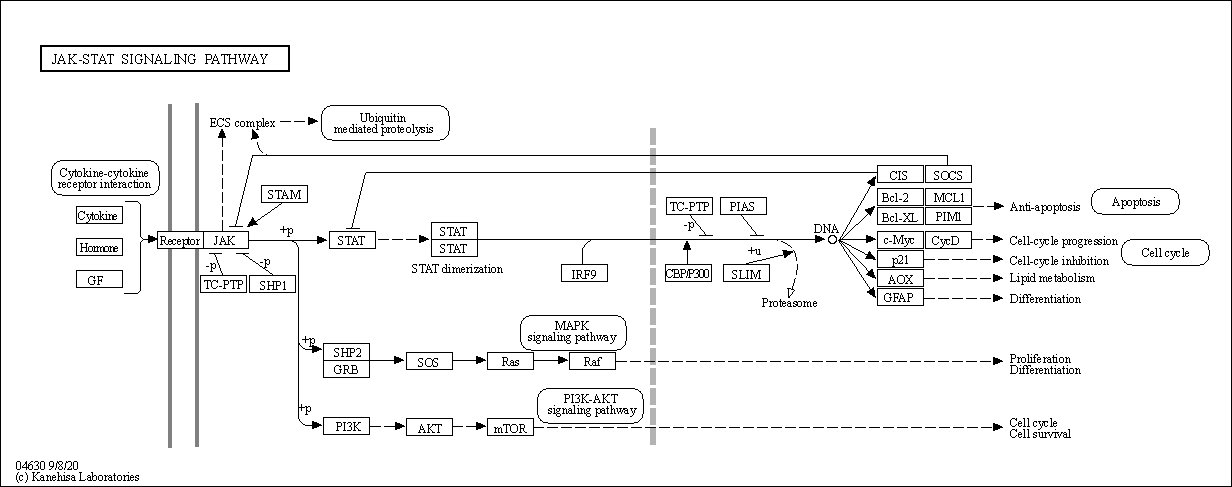

Supplement: Supplemental Information 3 [file peerj-09-11645-s003.png]
