# Supplementary material for: Analysis of ceRNA network of differentially expressed genes in FaDu cell line and a cisplatin-resistant line derived from it
Source: PeerJ. 2021 Jul 1;9:e11645. doi: 10.7717/peerj.11645 (PMC8255068; doi:10.7717/peerj.11645)
Supplement: Supplemental Information 4 [file peerj-09-11645-s004.docx]

Table S1 Abbreviations in this article

| Abbreviation | Full Name |
| --- | --- |
| RNA | ribonucleic acid |
| lncRNA | long non-coding RNA |
| circRNA | circular RNA |
| miRNA | micro RNA |
| ceRNA | competitive endogenous RNA |
| DEG | differentially expressed gene |
| GO | Gene Ontology |
| KEGG | Kyoto Encyclopedia of Genes and Genomes |
| DAVID | The Database for Annotation, Visualization and Integrated Discovery |
| RI | resistance index |
| IC50 | half inhibitory concentration |
| qRT-PCR | quantitative reverse transcription polymerase chain reaction |
| SD | standard deviation |
| EMT | epithelial-mesenchymal transition |
| ECM | extracellular matrix |
| CAF | cancer-associated fibroblast |
| MMP | matrix metalloproteinase |
| APP | beta-amyloid precursor protein |
| NPC | nasopharyngeal cancer |
| RCC | renal cell cancer |
| HCC | hepatocellular cancer |
