# Supplementary material for: Analysis of ceRNA network of differentially expressed genes in FaDu cell line and a cisplatin-resistant line derived from it
Source: PeerJ. 2021 Jul 1;9:e11645. doi: 10.7717/peerj.11645 (PMC8255068; doi:10.7717/peerj.11645)
Supplement: Supplemental Information 5 [file peerj-09-11645-s005.docx]

Table S2 Sequences of miRNA primers

| miRNA | sequence |
| --- | --- |
| miR-197-5p | 5’-TATACGGGTAGAGAGGGCAGTGG-3’ |
| miR-6840-3p | 5’-GCCCAGGACTTTGTGCGG-3’ |
| miR-6808-5p | 5’-CAGGCAGGGAGGTGGGA-3’ |
| miR-5006-5p | 5’-TTGCCAGGGCAGGAGGT-3’ |
| miR-7150 | 5’-CTGGCAGGGGGAGAGGTA-3’ |
| miR-34a-5p | 5’-CTGGCAGTGTCTTAGCTGGTTGT-3’ |
| miR-892b | 5’-CCACTGGCTCCTTTCTGGGTAG-3’ |
| miR-1229-5p | 5’-GTGGGTAGGGTTTGGGGGA-3’ |
