# Supplementary material for: Analysis of ceRNA network of differentially expressed genes in FaDu cell line and a cisplatin-resistant line derived from it
Source: PeerJ. 2021 Jul 1;9:e11645. doi: 10.7717/peerj.11645 (PMC8255068; doi:10.7717/peerj.11645)
Supplement: Supplemental Information 6 — Details of genes in ceRNA network. Gene names, gene types, their expression in FaDu and FaDu/DDP4, and fold-changes of these genes. [file peerj-09-11645-s006.docx]

Table S3 Expression and fold-changes of genes in ceRNA network

| Gene Name | Gene Type | Expression in  FaDu/DDP4 | Expression  in FaDu | Fold- Change |
| --- | --- | --- | --- | --- |
| *ACSL1* | mRNA | 11.876588 | 10.824656 | 2.0733042 |
| *ACTL7B* | mRNA | 3.0118793 | 7.412558 | 0.0473439 |
| *ANKRD22* | mRNA | 8.5659648 | 7.4885632 | 2.110232 |
| *APCDD1* | mRNA | 9.6637606 | 8.5913491 | 2.1029456 |
| *APOE* | mRNA | 8.6529802 | 10.160145 | 0.3518018 |
| *APOL6* | mRNA | 7.9738684 | 6.9085543 | 2.0926253 |
| *APPBP2* | mRNA | 7.0050676 | 5.5155862 | 2.8078802 |
| *ARHGAP18* | mRNA | 7.9690918 | 6.6177179 | 2.5515499 |
| *ATAD2* | mRNA | 9.6479532 | 8.59694 | 2.0719844 |
| *AVPR1A* | mRNA | 1.1411435 | 12.483636 | 0.0003851 |
| *BATF2* | mRNA | 8.5694532 | 7.4813641 | 2.1259227 |
| *BDP1* | mRNA | 7.5174103 | 6.3842844 | 2.1933347 |
| *BIRC3* | mRNA | 8.1070289 | 6.9720623 | 2.1961348 |
| *C1GALT1C1* | mRNA | 7.9838561 | 6.8135833 | 2.2505425 |
| *CA9* | mRNA | 6.0032949 | 7.0657339 | 0.4788219 |
| *CALCB* | mRNA | 10.517328 | 11.59549 | 0.4736319 |
| *CASP8AP2* | mRNA | 7.445639 | 6.3107178 | 2.1960657 |
| *CBR4* | mRNA | 7.8080214 | 6.7274566 | 2.1148638 |
| *CCDC88B* | mRNA | 7.7092444 | 6.5950428 | 2.1647518 |
| *CDA* | mRNA | 7.2586489 | 5.7807146 | 2.7854963 |
| *CEACAM6* | mRNA | 10.308691 | 8.203193 | 4.3034615 |
| *CEACAM7* | mRNA | 9.6420747 | 7.6072642 | 4.0976889 |
| *CENPE* | mRNA | 7.043666 | 6.0387722 | 2.0067958 |
| *CFAP74* | mRNA | 9.6170499 | 7.6386135 | 3.9406574 |
| *CHST1* | mRNA | 7.5414617 | 9.4904699 | 0.2589942 |
| *CMPK2* | mRNA | 13.732715 | 12.569854 | 2.2390098 |
| *CRELD2* | mRNA | 11.709306 | 13.043546 | 0.396601 |
| *CXCL10* | mRNA | 10.412744 | 8.2777691 | 4.3922943 |
| *CXCL11* | mRNA | 7.2618254 | 5.0395148 | 4.6664019 |
| *CYP24A1* | mRNA | 8.1891884 | 5.9739946 | 4.6434395 |
| *CYP4B1* | mRNA | 10.421379 | 9.3083675 | 2.1629673 |
| *DDX46* | mRNA | 7.0722602 | 6.0542521 | 2.025121 |
| *DDX60* | mRNA | 9.5582872 | 8.3556017 | 2.3016771 |
| *DDX60L* | mRNA | 7.3619294 | 6.0278441 | 2.521156 |
| *DIO2* | mRNA | 8.8366921 | 7.1731158 | 3.1680088 |
| *DKK3* | mRNA | 6.6182883 | 7.8696169 | 0.4200612 |
| *DPYD* | mRNA | 7.5597256 | 6.5012617 | 2.0827127 |
| *DPYSL5* | mRNA | 7.6907532 | 6.6352325 | 2.0784682 |
| *DYRK3* | mRNA | 7.5282184 | 12.816645 | 0.0255873 |
| *ECM2* | mRNA | 7.5677219 | 6.4795465 | 2.1260499 |
| *EOGT* | mRNA | 7.7534722 | 6.4062725 | 2.5441781 |
| *ERAP2* | mRNA | 10.723455 | 9.5811332 | 2.2073594 |
| *ERCC4* | mRNA | 7.2456294 | 5.3758498 | 3.6547675 |
| *ETV7* | mRNA | 12.082317 | 11.05048 | 2.0446264 |
| *F8* | mRNA | 9.7442457 | 8.6927042 | 2.0727433 |
| *FAM129A* | mRNA | 9.6326007 | 11.106285 | 0.3600617 |
| *FAS* | mRNA | 7.0615418 | 5.6614477 | 2.6391881 |
| *FOLH1* | mRNA | 7.5239539 | 5.8305984 | 3.2340805 |
| *FZD5* | mRNA | 9.3365641 | 8.2580034 | 2.111928 |
| *GABRP* | mRNA | 7.6851875 | 5.9727717 | 3.2770912 |
| *GBP1* | mRNA | 12.668497 | 11.595575 | 2.1036912 |
| *GBP4* | mRNA | 11.044024 | 9.8359224 | 2.3103336 |
| *GJA1* | mRNA | 7.7469619 | 6.5893191 | 2.2309262 |
| *GMPR* | mRNA | 8.8289559 | 7.7008669 | 2.1856904 |
| *GYPC* | mRNA | 2.0606347 | 7.2540907 | 0.0273284 |
| *HSPA5* | mRNA | 9.6768277 | 11.195531 | 0.3489996 |
| *HUNK* | mRNA | 4.4352585 | 11.072536 | 0.0100457 |
| *IFI44* | mRNA | 12.162821 | 11.116768 | 2.064873 |
| *IFI44L* | mRNA | 8.5372913 | 7.300153 | 2.3573047 |
| *IFIH1* | mRNA | 11.766531 | 10.385914 | 2.6037969 |
| *IFIT3* | mRNA | 14.067059 | 12.969108 | 2.1405042 |
| *IGF1* | mRNA | 1.1242637 | 7.1767104 | 0.0150672 |
| *IKZF2* | mRNA | 7.3207807 | 5.6180475 | 3.2551706 |
| *INPP5D* | mRNA | 7.5637042 | 6.4734567 | 2.1291057 |
| *IRF5* | mRNA | 8.1859509 | 7.1274376 | 2.0827842 |
| *KDELC2* | mRNA | 8.1358238 | 7.0517781 | 2.1199728 |
| *KIAA0922* | mRNA | 8.4553898 | 12.424296 | 0.0638616 |
| *KLHDC7B* | mRNA | 8.4988476 | 10.047423 | 0.3418475 |
| *KLK9* | mRNA | 7.2631772 | 8.6182263 | 0.3909215 |
| *KRT27* | mRNA | 7.4842015 | 5.2092544 | 4.8397991 |
| *KRT35* | mRNA | 3.5184839 | 7.9539704 | 0.0462153 |
| *LCT* | mRNA | 9.0334723 | 10.139366 | 0.4646145 |
| *LGALS7* | mRNA | 9.3075602 | 10.597907 | 0.4088527 |
| *MANF* | mRNA | 11.555543 | 12.70755 | 0.4499986 |
| *MAP9* | mRNA | 7.1010635 | 5.7064878 | 2.6291121 |
| *MBLAC2* | mRNA | 7.3348614 | 6.3234777 | 2.0158436 |
| *METTL7A* | mRNA | 8.2992634 | 7.2842914 | 2.0208637 |
| *MGLL* | mRNA | 8.000996 | 9.1013634 | 0.4663977 |
| *MMP1* | mRNA | 11.054805 | 9.9532961 | 2.1457905 |
| *MMP13* | mRNA | 9.3943427 | 7.84535 | 2.9261278 |
| *MSX1* | mRNA | 8.2674353 | 6.8113674 | 2.7435957 |
| *NBN* | mRNA | 9.2803172 | 7.9462252 | 2.5211677 |
| *NEXN* | mRNA | 8.3061035 | 6.9025206 | 2.6455778 |
| *NUDCD1* | mRNA | 7.4971463 | 6.3237946 | 2.2553506 |
| *NUDT12* | mRNA | 8.4670013 | 7.3150883 | 2.2220835 |
| *NXPH4* | mRNA | 8.1674809 | 9.2029889 | 0.4878441 |
| *OR13F1* | mRNA | 1.4229561 | 8.8703281 | 0.0057295 |
| *OR13J1* | mRNA | 3.0405943 | 12.058771 | 0.0019287 |
| *OTX2* | mRNA | 1.164972 | 7.9216109 | 0.009248 |
| *PARD6A* | mRNA | 10.324598 | 11.770199 | 0.3671392 |
| *PCGF5* | mRNA | 10.500563 | 9.7655957 | 1.6643598 |
| *PM20D2* | mRNA | 7.7998534 | 6.791027 | 2.0122735 |
| *PPAP2B* | mRNA | 9.4081054 | 8.3186526 | 2.1279331 |
| *PPP6R2* | mRNA | 7.471997 | 6.4355167 | 2.0512173 |
| *PRR15L* | mRNA | 8.0603719 | 5.736667 | 5.0061616 |
| *PRSS1* | mRNA | 5.9798206 | 7.2150006 | 0.4247895 |
| *PTPN20* | mRNA | 1.2399473 | 10.739177 | 0.0013818 |
| *RABGAP1L* | mRNA | 9.2589261 | 8.2277844 | 2.0436408 |
| *RASSF6* | mRNA | 7.9298133 | 6.6279089 | 2.4655413 |
| *RB1CC1* | mRNA | 7.6238842 | 6.6161704 | 2.0107221 |
| *RFX7* | mRNA | 7.0012532 | 5.8405888 | 2.2356036 |
| *RUNDC3A* | mRNA | 7.4320714 | 8.4898113 | 0.480384 |
| *S100A9* | mRNA | 13.020092 | 10.954435 | 4.1862471 |
| *SAMD9* | mRNA | 10.644944 | 9.2255944 | 2.6746481 |
| *SAMD9L* | mRNA | 8.0712206 | 6.5619853 | 2.8465913 |
| *SASH1* | mRNA | 10.826675 | 9.7447341 | 2.1168822 |
| *SCTR* | mRNA | 1.8370588 | 14.058667 | 0.0002094 |
| *SERPINA3* | mRNA | 7.3040111 | 8.3634928 | 0.4798044 |
| *SHC2* | mRNA | 6.1160385 | 7.7058093 | 0.3322242 |
| *SLC22A8* | mRNA | 7.5288036 | 4.4418086 | 8.4972443 |
| *SLC27A2* | mRNA | 8.5153542 | 7.4958905 | 2.0271654 |
| *SLCO2A1* | mRNA | 6.4019982 | 7.7044948 | 0.405424 |
| *SLITRK4* | mRNA | 1.6454247 | 10.385529 | 0.0023387 |
| *SMCHD1* | mRNA | 7.2161725 | 5.7253489 | 2.8104938 |
| *SMCO2* | mRNA | 4.5398853 | 15.208113 | 0.0006145 |
| *SOS1* | mRNA | 7.1717026 | 6.0738 | 2.1404329 |
| *SOWAHD* | mRNA | 7.01557 | 5.8487428 | 2.245174 |
| *SPATA16* | mRNA | 1.4223945 | 13.063335 | 0.0003131 |
| *SPRR1B* | mRNA | 9.8280951 | 8.4043894 | 2.6827371 |
| *SPRR2A* | mRNA | 7.7009478 | 6.3262885 | 2.5930666 |
| *SPRR2B* | mRNA | 7.4049917 | 5.7480188 | 3.1535413 |
| *SPRR2D* | mRNA | 7.9351647 | 6.5205838 | 2.6658227 |
| *SPRR2F* | mRNA | 7.0878694 | 5.9284894 | 2.2336142 |
| *ST6GAL1* | mRNA | 8.172837 | 9.4069182 | 0.4251132 |
| *STMN3* | mRNA | 11.285553 | 9.9511532 | 2.5217053 |
| *TANC2* | mRNA | 7.0160535 | 8.0233428 | 0.4974801 |
| *TC2N* | mRNA | 7.6920845 | 3.9074477 | 13.781268 |
| *TEKT4* | mRNA | 8.80929 | 9.9368522 | 0.4576884 |
| *TET2* | mRNA | 7.775704 | 6.7612259 | 2.020172 |
| *TIMP4* | mRNA | 7.2029946 | 8.8623024 | 0.316591 |
| *TLR3* | mRNA | 8.3964033 | 6.9286056 | 2.7659933 |
| *TM4SF1* | mRNA | 10.913564 | 9.4124588 | 2.8305955 |
| *TMEM171* | mRNA | 7.0172286 | 12.293823 | 0.025798 |
| *TNFAIP8* | mRNA | 9.6971538 | 8.6570004 | 2.0564463 |
| *TNNT1* | mRNA | 2.1380131 | 12.296593 | 0.0008749 |
| *TRIM22* | mRNA | 12.569854 | 10.984764 | 3.0002656 |
| *TRIM5* | mRNA | 7.7692227 | 6.7105446 | 2.0830221 |
| *TWSG1* | mRNA | 8.3197582 | 7.1012579 | 2.327047 |
| *TXNIP* | mRNA | 13.908456 | 10.550587 | 10.252255 |
| *TYMSOS* | mRNA | 9.8284167 | 8.6409796 | 2.277478 |
| *TYW3* | mRNA | 7.6229133 | 6.5853105 | 2.0528138 |
| *UFL1* | mRNA | 7.93226 | 6.9049653 | 2.0381987 |
| *UPF2* | mRNA | 9.3656857 | 8.3203205 | 2.0638886 |
| *USH1G* | mRNA | 6.6539302 | 8.3542367 | 0.3077207 |
| *VAV3* | mRNA | 8.792085 | 7.1721878 | 3.0735313 |
| *VCPIP1* | mRNA | 8.0281628 | 6.9935589 | 2.0485512 |
| *VPS13A* | mRNA | 7.8994296 | 6.6686608 | 2.3469203 |
| *WFIKKN2* | mRNA | 3.6606689 | 7.9705237 | 0.0504202 |
| *ZBP1* | mRNA | 9.4132051 | 7.8620713 | 2.9304734 |
| *ZNF358* | mRNA | 12.32402 | 13.349189 | 0.4913527 |
| *ZNF414* | mRNA | 5.1676849 | 7.2556537 | 0.2352116 |
| *ZNF518A* | mRNA | 7.0716551 | 6.0415353 | 2.0421938 |
| *ZNF568* | mRNA | 4.0142032 | 13.44776 | 0.0014462 |
| *ZNF628* | mRNA | 9.0039463 | 10.46379 | 0.3635326 |
| *ZNF792* | mRNA | 7.3088211 | 5.1546985 | 4.4509786 |
| *NR_003261* | lncRNA | 1.4093685 | 12.692812 | 0.0004012 |
| *NR_027454* | lncRNA | 5.0725173 | 15.34646 | 0.0008077 |
| *NR_027455* | lncRNA | 1.6547352 | 7.2631524 | 0.0204974 |
| *NR_028326* | lncRNA | 8.9919171 | 13.746051 | 0.0370564 |
| *NR_036501* | lncRNA | 1.1180046 | 7.3154539 | 0.0136264 |
| *NR_038328* | lncRNA | 8.8811631 | 10.065292 | 0.4400902 |
| *NR_038446* | lncRNA | 7.0906513 | 1.3305127 | 54.196906 |
| *NR_103538* | lncRNA | 8.2736457 | 6.5962035 | 3.1986035 |
| *NR_104598* | lncRNA | 9.6723559 | 8.6506815 | 2.0302739 |
| *NR_106723* | lncRNA | 6.7479754 | 11.525854 | 0.0364515 |
| *NR_109986* | lncRNA | 1.126743 | 7.028761 | 0.0167231 |
| *NR_110199* | lncRNA | 2.4117176 | 11.690353 | 0.0016101 |
| *NR_111911* | lncRNA | 1.4019552 | 10.461143 | 0.0018746 |
| *NR_125395* | lncRNA | 4.8717512 | 7.0414624 | 0.2222552 |
| *lnc-ABCD3-1:2* | lncRNA | 9.1109406 | 8.0220744 | 2.127068 |
| *lnc-ACAD11-2:1* | lncRNA | 2.1949888 | 7.3927473 | 0.027247 |
| *lnc-BANP-7:1* | lncRNA | 2.6530725 | 7.0481257 | 0.0475288 |
| *lnc-BHLHA15-1:1* | lncRNA | 5.9295902 | 7.6844398 | 0.2963041 |
| *lnc-CXCL3-1:1* | lncRNA | 7.7670542 | 6.3135 | 2.7388195 |
| *lnc-DMRTA1-22:1* | lncRNA | 2.1626895 | 7.7275618 | 0.0211255 |
| *lnc-DONSON-2:1* | lncRNA | 6.9681756 | 8.0267632 | 0.4801019 |
| *lnc-EFNB1-2:1* | lncRNA | 1.2548325 | 8.8218743 | 0.0052734 |
| *lnc-GPAT2-2:1* | lncRNA | 1.2480373 | 10.929731 | 0.0012176 |
| *lnc-GRIA3-1:3* | lncRNA | 6.2189127 | 7.3060544 | 0.470693 |
| *lnc-ILK-8:1* | lncRNA | 1.1474317 | 12.067773 | 0.000516 |
| *lnc-LIN28B-5:1* | lncRNA | 6.9022356 | 8.2416905 | 0.3951699 |
| *lnc-MACF1-2:1* | lncRNA | 1.4021901 | 12.49769 | 0.000457 |
| *lnc-MORF4L1-4:2* | lncRNA | 7.0387014 | 5.2187996 | 3.5305715 |
| *lnc-MREG-3:1* | lncRNA | 6.045384 | 7.4415272 | 0.3799435 |
| *lnc-MYLPF-3:1* | lncRNA | 5.1237172 | 7.0882667 | 0.2562192 |
| *lnc-NCOA3-4:1* | lncRNA | 2.844293 | 12.017582 | 0.0017321 |
| *lnc-NRIP1-5:1* | lncRNA | 1.7134873 | 7.1218343 | 0.0235464 |
| *lnc-NSRP1-3:1* | lncRNA | 5.2181971 | 13.27195 | 0.0037634 |
| *lnc-OR4A16-1:1* | lncRNA | 1.3609173 | 7.1337926 | 0.0182891 |
| *lnc-PLEKHA7-2:1* | lncRNA | 9.5866514 | 8.542377 | 2.0623288 |
| *lnc-RAB15-2:5* | lncRNA | 7.0544336 | 6.0250927 | 2.0410914 |
| *lnc-RBMS1-7:1* | lncRNA | 6.3555757 | 8.2809432 | 0.2632732 |
| *lnc-RHOD-2:1* | lncRNA | 5.3508367 | 12.23938 | 0.00844 |
| *lnc-SERP1-2:1* | lncRNA | 8.8399962 | 12.965883 | 0.0572776 |
| *lnc-SETMAR-2:1* | lncRNA | 7.3546238 | 5.6057938 | 3.3608589 |
| *lnc-SIX1-1:1* | lncRNA | 3.3839204 | 8.9981067 | 0.0204156 |
| *lnc-SLC1A1-7:1* | lncRNA | 5.1498246 | 12.653173 | 0.0055115 |
| *lnc-SORCS1-5:1* | lncRNA | 1.1394542 | 7.177948 | 0.0152136 |
| *lnc-SPTLC1-3:1* | lncRNA | 1.1010673 | 14.643896 | 8.379E-05 |
| *lnc-TCP10-3:1* | lncRNA | 1.4948646 | 13.807442 | 0.0001966 |
| *lnc-TFB2M-5:1* | lncRNA | 5.6753931 | 7.0667393 | 0.3812089 |
| *lnc-TOP3A-2:1* | lncRNA | 4.5784259 | 7.371682 | 0.1442601 |
| *lnc-TPGS2-3:2* | lncRNA | 7.860417 | 6.6337729 | 2.3402199 |
| *lnc-VPS36-2:1* | lncRNA | 7.9172064 | 6.7574331 | 2.2342231 |
| *lnc-YIF1B-1:1* | lncRNA | 6.0952949 | 7.1378252 | 0.4854753 |
| *lnc-ZDHHC11-1:1* | lncRNA | 5.1542008 | 9.2327405 | 0.0591885 |
| *lnc-ZMYM3-3:1* | lncRNA | 4.8896746 | 12.304436 | 0.0058605 |
| *hsa_circ_0001784* | circRNA | 5.2812288 | 7.5314756 | 0.2101881 |
| *hsa_circ_0002936* | circRNA | 6.6460534 | 10.737884 | 0.0586457 |
| *hsa_circ_0004646* | circRNA | 4.1258398 | 8.9969112 | 0.0341713 |
| *hsa_circ_0005064* | circRNA | 5.5312047 | 10.983306 | 0.022843 |
| *hsa_circ_0005721* | circRNA | 2.6553026 | 7.2475665 | 0.0414563 |
| *hsa_circ_0006113* | circRNA | 5.2010406 | 7.0520583 | 0.2771968 |
| *hsa_circ_0007260* | circRNA | 7.3768579 | 3.0814815 | 19.635281 |
| *hsa_circ_0007371* | circRNA | 7.2464822 | 1.1064274 | 70.524607 |
| *hsa_circ_0007377* | circRNA | 5.7235197 | 7.7524183 | 0.2450421 |
| *hsa_circ_0007596* | circRNA | 1.2374811 | 13.673647 | 0.0001804 |
| *hsa_circ_0009546* | circRNA | 2.8538537 | 9.7822245 | 0.0082102 |
| *hsa_circ_0009836* | circRNA | 5.549265 | 7.1027512 | 0.3406858 |
| *hsa_circ_0010395* | circRNA | 6.5822259 | 11.85298 | 0.0259027 |
| *hsa_circ_0010657* | circRNA | 4.3748188 | 12.120417 | 0.0046595 |
| *hsa_circ_0011780* | circRNA | 5.7233225 | 16.994603 | 0.0004046 |
| *hsa_circ_0012109* | circRNA | 3.9460608 | 13.652243 | 0.0011971 |
| *hsa_circ_0013013* | circRNA | 8.9096614 | 7.7071026 | 2.3014751 |
| *hsa_circ_0013809* | circRNA | 2.5076636 | 7.7741156 | 0.02598 |
| *hsa_circ_0013881* | circRNA | 10.161766 | 6.8908394 | 9.6526597 |
| *hsa_circ_0013882* | circRNA | 9.0866675 | 5.854112 | 9.3993137 |
| *hsa_circ_0013883* | circRNA | 9.9626327 | 6.386309 | 11.928359 |
| *hsa_circ_0014220* | circRNA | 11.760112 | 9.7046976 | 4.1566312 |
| *hsa_circ_0014221* | circRNA | 10.372563 | 8.4595991 | 3.7658194 |
| *hsa_circ_0016686* | circRNA | 7.9788281 | 6.2502139 | 3.3140932 |
| *hsa_circ_0017886* | circRNA | 2.5926654 | 9.2826649 | 0.0096852 |
| *hsa_circ_0018338* | circRNA | 6.2511124 | 7.6104065 | 0.3897729 |
| *hsa_circ_0018367* | circRNA | 7.2340404 | 8.5906767 | 0.3904917 |
| *hsa_circ_0019547* | circRNA | 3.0216511 | 10.453887 | 0.0057899 |
| *hsa_circ_0021215* | circRNA | 4.7516738 | 7.3063634 | 0.1702009 |
| *hsa_circ_0021487* | circRNA | 5.0883654 | 7.8682694 | 0.1456014 |
| *hsa_circ_0021580* | circRNA | 4.8013065 | 7.1629227 | 0.1945731 |
| *hsa_circ_0022993* | circRNA | 7.2807596 | 6.2616591 | 2.026655 |
| *hsa_circ_0023300* | circRNA | 12.806392 | 11.70374 | 2.1474911 |
| *hsa_circ_0023372* | circRNA | 7.8755184 | 9.7796434 | 0.2671784 |
| *hsa_circ_0024522* | circRNA | 6.7095076 | 7.7822013 | 0.4754305 |
| *hsa_circ_0024523* | circRNA | 7.0996865 | 8.1724608 | 0.4754039 |
| *hsa_circ_0025801* | circRNA | 3.3137361 | 12.056167 | 0.0023349 |
| *hsa_circ_0025968* | circRNA | 7.1059804 | 2.7322453 | 20.731249 |
| *hsa_circ_0026298* | circRNA | 6.8949398 | 13.157348 | 0.0130265 |
| *hsa_circ_0026477* | circRNA | 10.337993 | 9.3202291 | 2.0247779 |
| *hsa_circ_0027747* | circRNA | 7.2597994 | 4.7621651 | 5.6475862 |
| *hsa_circ_0027755* | circRNA | 5.4256635 | 9.5291834 | 0.0581725 |
| *hsa_circ_0028223* | circRNA | 7.2273971 | 1.1146589 | 69.201827 |
| *hsa_circ_0029317* | circRNA | 6.8005466 | 8.104927 | 0.404895 |
| *hsa_circ_0030784* | circRNA | 7.5879677 | 6.4129928 | 2.2578895 |
| *hsa_circ_0030845* | circRNA | 1.1248079 | 7.0484356 | 0.0164744 |
| *hsa_circ_0031002* | circRNA | 7.7469935 | 8.923987 | 0.4422722 |
| *hsa_circ_0031285* | circRNA | 7.8340825 | 6.7854372 | 2.0685865 |
| *hsa_circ_0031478* | circRNA | 2.1924234 | 15.567879 | 9.41E-05 |
| *hsa_circ_0031501* | circRNA | 7.0770578 | 3.9342548 | 8.8323847 |
| *hsa_circ_0034241* | circRNA | 5.6789265 | 8.1829748 | 0.1762813 |
| *hsa_circ_0034434* | circRNA | 3.8830261 | 7.727427 | 0.0696178 |
| *hsa_circ_0034976* | circRNA | 5.8648348 | 7.2647366 | 0.3789549 |
| *hsa_circ_0035263* | circRNA | 5.2853033 | 7.4793416 | 0.2185389 |
| *hsa_circ_0035745* | circRNA | 4.4302692 | 7.2661056 | 0.1400645 |
| *hsa_circ_0036442* | circRNA | 4.010326 | 11.415342 | 0.0059002 |
| *hsa_circ_0036451* | circRNA | 8.0345657 | 6.3913547 | 3.1236027 |
| *hsa_circ_0037880* | circRNA | 7.1078155 | 17.067223 | 0.0010044 |
| *hsa_circ_0037947* | circRNA | 1.1168298 | 7.8542766 | 0.0093719 |
| *hsa_circ_0038079* | circRNA | 8.2093008 | 6.1588238 | 4.1424291 |
| *hsa_circ_0039040* | circRNA | 1.2409969 | 11.428737 | 0.0008574 |
| *hsa_circ_0039306* | circRNA | 8.0292379 | 6.4818585 | 2.9228574 |
| *hsa_circ_0039561* | circRNA | 1.1098618 | 8.9881049 | 0.0042502 |
| *hsa_circ_0041393* | circRNA | 8.1275734 | 5.6775885 | 5.4641041 |
| *hsa_circ_0041823* | circRNA | 7.3136876 | 5.4791756 | 3.5665074 |
| *hsa_circ_0041981* | circRNA | 12.515059 | 8.0822213 | 21.598183 |
| *hsa_circ_0042611* | circRNA | 6.6483934 | 7.6551636 | 0.4976591 |
| *hsa_circ_0042782* | circRNA | 6.8260332 | 8.0074923 | 0.4409053 |
| *hsa_circ_0042905* | circRNA | 7.4380095 | 1.122873 | 79.624276 |
| *hsa_circ_0043158* | circRNA | 8.2269368 | 6.6816478 | 2.9186254 |
| *hsa_circ_0043298* | circRNA | 3.4298579 | 12.149367 | 0.0023723 |
| *hsa_circ_0043546* | circRNA | 9.0574907 | 8.0420621 | 2.0215034 |
| *hsa_circ_0044998* | circRNA | 5.1813079 | 8.3307078 | 0.1127032 |
| *hsa_circ_0045077* | circRNA | 5.6650869 | 12.096031 | 0.0115903 |
| *hsa_circ_0045753* | circRNA | 10.392986 | 13.595851 | 0.108603 |
| *hsa_circ_0046671* | circRNA | 9.0327915 | 7.997318 | 2.0497862 |
| *hsa_circ_0046726* | circRNA | 7.0788108 | 5.7638307 | 2.487989 |
| *hsa_circ_0047512* | circRNA | 7.1201179 | 5.9820104 | 2.2009213 |
| *hsa_circ_0047537* | circRNA | 3.5109893 | 8.4916188 | 0.0316724 |
| *hsa_circ_0047589* | circRNA | 5.1336482 | 7.6648926 | 0.1729894 |
| *hsa_circ_0049636* | circRNA | 10.978119 | 11.994988 | 0.4941878 |
| *hsa_circ_0049638* | circRNA | 10.136243 | 11.200984 | 0.4780584 |
| *hsa_circ_0049639* | circRNA | 9.1042648 | 10.191368 | 0.4707056 |
| *hsa_circ_0049640* | circRNA | 11.326906 | 12.357702 | 0.4894401 |
| *hsa_circ_0049641* | circRNA | 11.300961 | 12.332539 | 0.489175 |
| *hsa_circ_0049642* | circRNA | 10.435337 | 11.505109 | 0.476394 |
| *hsa_circ_0049980* | circRNA | 4.5657661 | 9.1784641 | 0.0408733 |
| *hsa_circ_0050809* | circRNA | 5.3909414 | 7.4053052 | 0.2475233 |
| *hsa_circ_0052161* | circRNA | 7.1609125 | 4.9191212 | 4.7298395 |
| *hsa_circ_0052946* | circRNA | 1.0892581 | 7.7827246 | 0.009662 |
| *hsa_circ_0053766* | circRNA | 8.429883 | 5.0134764 | 10.676794 |
| *hsa_circ_0054056* | circRNA | 5.043631 | 15.614895 | 0.0006573 |
| *hsa_circ_0054181* | circRNA | 5.9883922 | 7.5963252 | 0.328068 |
| *hsa_circ_0054541* | circRNA | 10.806896 | 4.6673693 | 70.498792 |
| *hsa_circ_0054763* | circRNA | 3.2006538 | 7.7954743 | 0.0413829 |
| *hsa_circ_0054801* | circRNA | 5.741965 | 17.302949 | 0.000331 |
| *hsa_circ_0058265* | circRNA | 5.9065289 | 11.928883 | 0.0153848 |
| *hsa_circ_0058266* | circRNA | 5.7595908 | 8.1106982 | 0.1959955 |
| *hsa_circ_0059159* | circRNA | 5.3246847 | 7.2536307 | 0.262621 |
| *hsa_circ_0059873* | circRNA | 7.525329 | 6.3432849 | 2.2689802 |
| *hsa_circ_0060418* | circRNA | 10.583471 | 12.254972 | 0.3139266 |
| *hsa_circ_0060939* | circRNA | 7.5959646 | 5.9120394 | 3.2130093 |
| *hsa_circ_0061865* | circRNA | 6.4780659 | 8.2080415 | 0.3014571 |
| *hsa_circ_0062235* | circRNA | 7.4296026 | 9.7411312 | 0.2014469 |
| *hsa_circ_0062458* | circRNA | 4.6505509 | 7.1356925 | 0.1786067 |
| *hsa_circ_0064840* | circRNA | 7.3629488 | 1.1889788 | 72.202156 |
| *hsa_circ_0065114* | circRNA | 12.674116 | 1.4046881 | 2468.516 |
| *hsa_circ_0065115* | circRNA | 4.4716059 | 12.110946 | 0.0050157 |
| *hsa_circ_0065191* | circRNA | 9.7649864 | 12.660809 | 0.1343602 |
| *hsa_circ_0065239* | circRNA | 2.1948363 | 9.9344153 | 0.004679 |
| *hsa_circ_0065315* | circRNA | 4.022804 | 11.232652 | 0.0067549 |
| *hsa_circ_0065398* | circRNA | 8.0255026 | 11.380205 | 0.0977538 |
| *hsa_circ_0065610* | circRNA | 7.7201083 | 5.4774269 | 4.7327588 |
| *hsa_circ_0068269* | circRNA | 5.665217 | 10.549277 | 0.033865 |
| *hsa_circ_0068444* | circRNA | 7.3056973 | 8.3340967 | 0.4902538 |
| *hsa_circ_0070317* | circRNA | 4.430841 | 9.9432955 | 0.0219071 |
| *hsa_circ_0071595* | circRNA | 7.40237 | 12.16214 | 0.0369119 |
| *hsa_circ_0071622* | circRNA | 7.1958535 | 1.1017619 | 68.313158 |
| *hsa_circ_0072737* | circRNA | 6.0860733 | 8.8192982 | 0.1503894 |
| *hsa_circ_0073598* | circRNA | 3.8870427 | 8.7496723 | 0.0343718 |
| *hsa_circ_0074928* | circRNA | 7.5365746 | 1.1203966 | 85.40082 |
| *hsa_circ_0075482* | circRNA | 4.0635193 | 7.2781171 | 0.1077233 |
| *hsa_circ_0075838* | circRNA | 2.264443 | 7.8016478 | 0.0215345 |
| *hsa_circ_0077117* | circRNA | 8.2604898 | 5.3556386 | 7.4894055 |
| *hsa_circ_0077139* | circRNA | 5.6241798 | 7.8541445 | 0.2131639 |
| *hsa_circ_0077308* | circRNA | 4.4625558 | 7.7975645 | 0.0990974 |
| *hsa_circ_0078455* | circRNA | 5.0300138 | 7.9344275 | 0.1335624 |
| *hsa_circ_0079194* | circRNA | 5.8193426 | 9.258696 | 0.0921831 |
| *hsa_circ_0080280* | circRNA | 6.9900052 | 7.9967072 | 0.4976827 |
| *hsa_circ_0081123* | circRNA | 3.5659651 | 8.2973674 | 0.0376449 |
| *hsa_circ_0081463* | circRNA | 5.8035054 | 10.434761 | 0.0403509 |
| *hsa_circ_0083301* | circRNA | 5.8150672 | 11.161594 | 0.0245773 |
| *hsa_circ_0083521* | circRNA | 6.0685448 | 8.2956239 | 0.2135907 |
| *hsa_circ_0083665* | circRNA | 5.3313209 | 7.1008887 | 0.2932966 |
| *hsa_circ_0083666* | circRNA | 5.9122355 | 7.4619045 | 0.3415884 |
| *hsa_circ_0083667* | circRNA | 5.3222499 | 7.2101018 | 0.2702091 |
| *hsa_circ_0083668* | circRNA | 5.596791 | 7.1845521 | 0.3326873 |
| *hsa_circ_0084420* | circRNA | 6.9948991 | 12.277759 | 0.0256863 |
| *hsa_circ_0085230* | circRNA | 6.9393336 | 13.025847 | 0.0147156 |
| *hsa_circ_0085645* | circRNA | 8.0938285 | 9.3786789 | 0.4104134 |
| *hsa_circ_0087726* | circRNA | 3.961836 | 7.1215456 | 0.1119007 |
| *hsa_circ_0088326* | circRNA | 4.2574228 | 7.0494079 | 0.1443872 |
| *hsa_circ_0088564* | circRNA | 8.8781062 | 10.388691 | 0.3509689 |
| *hsa_circ_0089656* | circRNA | 7.5688867 | 6.1904696 | 2.5998296 |
| *hsa_circ_0089771* | circRNA | 5.1027235 | 11.467088 | 0.0121377 |
| *hsa_circ_0090158* | circRNA | 7.4427909 | 3.9776722 | 11.043447 |
| *hsa_circ_0092149* | circRNA | 7.6911333 | 1.0919124 | 96.953489 |
| *hsa_circ_0092241* | circRNA | 1.1039743 | 11.718173 | 0.000638 |
| *hsa-miR-1224-5p* | miRNA | 3.3354933 | 2.6958448 | 1.5579494 |
| *hsa-miR-1229-5p* | miRNA | 4.0888641 | 3.1063005 | 1.9759735 |
| *hsa-miR-128-3p* | miRNA | 3.504593 | 2.9174271 | 1.5022926 |
| *hsa-miR-1288-3p* | miRNA | 4.0665529 | 3.477324 | 1.5044425 |
| *hsa-miR-197-5p* | miRNA | 6.7507613 | 7.4373469 | 0.6213226 |
| *hsa-miR-221-5p* | miRNA | 3.5660301 | 2.9661756 | 1.5155637 |
| *hsa-miR-30a-5p* | miRNA | 5.1986615 | 4.4102972 | 1.7271151 |
| *hsa-miR-34a-5p* | miRNA | 4.3958611 | 3.5036943 | 1.8559615 |
| *hsa-miR-4672* | miRNA | 3.8453879 | 3.0307233 | 1.7588892 |
| *hsa-miR-4787-5p* | miRNA | 3.7838869 | 3.1304849 | 1.5728728 |
| *hsa-miR-5006-5p* | miRNA | 4.0434861 | 3.4389699 | 1.5204687 |
| *hsa-miR-6076* | miRNA | 3.6641166 | 2.8902384 | 1.70986 |
| *hsa-miR-6808-5p* | miRNA | 4.3003588 | 3.6771913 | 1.5402532 |
| *hsa-miR-6840-3p* | miRNA | 3.438718 | 2.8322244 | 1.5225542 |
| *hsa-miR-6887-5p* | miRNA | 3.6521271 | 2.9817013 | 1.5915426 |
| *hsa-miR-7150* | miRNA | 3.4828472 | 2.8323935 | 1.5696618 |
| *hsa-miR-8063* | miRNA | 3.7815388 | 3.0456313 | 1.6654447 |
| *hsa-miR-892b* | miRNA | 3.636575 | 3.0268378 | 1.5259812 |
