# Supplementary material for: Analysis of ceRNA network of differentially expressed genes in FaDu cell line and a cisplatin-resistant line derived from it
Source: PeerJ. 2021 Jul 1;9:e11645. doi: 10.7717/peerj.11645 (PMC8255068; doi:10.7717/peerj.11645)

**Miame Checklist**

Part 1 Experiment description

-    **Human hypopharyngeal cancer cell, FaDu**

-        **experimental variables (parent cell, FaDu vs. cisplatin-resistant cell, FaDu/DDP4)**

-        **n-count for each group is 1**

Part 2 Array design.

-       **Agilent SurePrint G3 Microarray – Human, SBC Human (4*180K) ceRNA Microarray and Agilent Human (8*60K) miRNA Microarray**

-         **Array type (human, ceRNA and miRNA)**

-        **18853 probes for mRNA, 77103 probes for lncRNA, 88371 probes for circRNA, 2549 probes for miRNA**

-         **Each probe for mRNA, lncRNA and circRNA was 60 mer long**

Part 3 Samples

-         **Cy3 labels for cells**

-         **There are dye controls and quality control probes in the microarray.**

-        **Total RNA was labeled by Low Input Quick Amp Labeling Kit, One-Color (Cat.# 5190-2305, Agilent technologies, Santa Clara, CA, US), following the manufacturer’s instructions.**

-        **Total RNA was extracted and purified using miRNeasy Mini Kit（Cat#217004，QIAGEN, GmBH, Germany) following the manufacturer’s instructions and checked for a RIN number to inspect RNA integration by an Agilent Bioanalyzer 2100 (Agilent technologies, Santa Clara, CA, US).**

Part 4 Hybridizations

-        **Each slide was hybridized with 1.65μg Cy3-labeled cRNA using Gene Expression Hybridization Kit (Cat.# 5188-5242, Agilent technologies, Santa Clara, CA, US) in Hybridization Oven (Cat.# G2545A, Agilent technologies, Santa Clara, CA, US), according to the manufacturer’s instructions. After 17 hours hybridization, slides were washed in staining dishes (Cat.# 121, Thermo Shandon, Waltham, MA, US) with Gene Expression Wash Buffer Kit(Cat.# 5188-5327, Agilent technologies, Santa Clara, CA, US), followed the manufacturer’s instructions**

-        **Blocking buffer: 10X Blocking Agent (Agilent p/n 5188-5281) diluted to 1X by nuclease-free water**

-       **Hybridization Buffer: 2x GEx Hybridization Buffer HI-RPM**

-       **wash buffer:** **0.005% Triton X-102 in the Gene Expression wash buffers**

Part 5 Measurements

-         **Slides were scanned by Agilent Microarray Scanner (Cat#G2565CA, Agilent technologies, Santa Clara, CA, US) with default settings, Dye channel: Green, Scan resolution=3μm, PMT 100%, 20bit. Data were extracted with Feature Extraction software 10.7 (Agilent technologies, Santa Clara, CA, US). Raw data were normalized by Quantile algorithm, limma packages in R.**

-         **There are several repeat quality control probes in microarray to calculate CV value to assess the stability of microarray.**

Part 6 Normalization controls

 -        **The original scanned chip data is normalized by the limma package in R. The algorithm used is Quantile, and the normalized signal value is the signal value calculated by log2.**

  -        **When comparing data across a set of one-color microarrays, a simple linear scaling of the data is usually sufficient for most experimental applications. As Agilent determined, use the 75th percentile signal value of all of non-control probes to normalize Agilent one-color microarray signals for inter-array comparisons.**

**Figure 2 FaDu/DDP4 - miRNA Microarray**


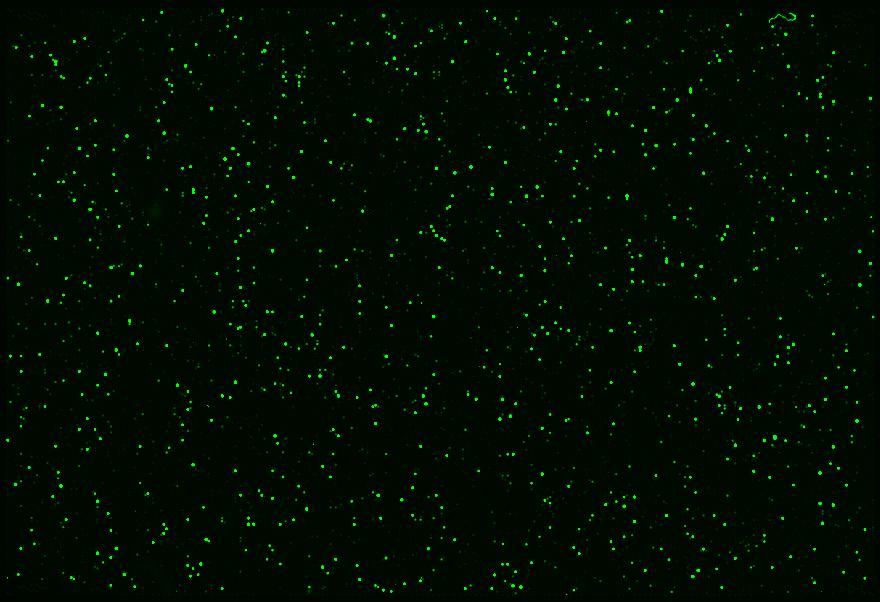


**Figure 1 FaDu - miRNA Microarray**


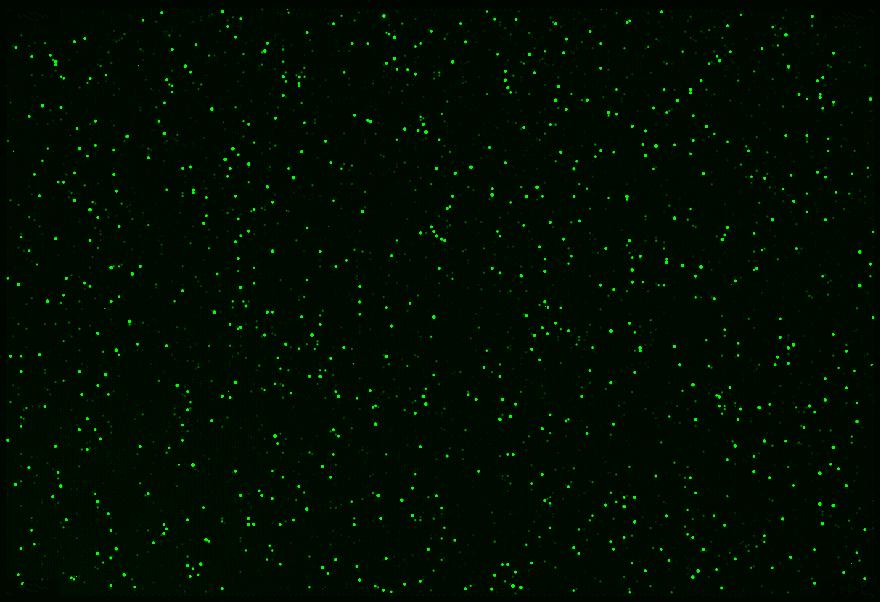

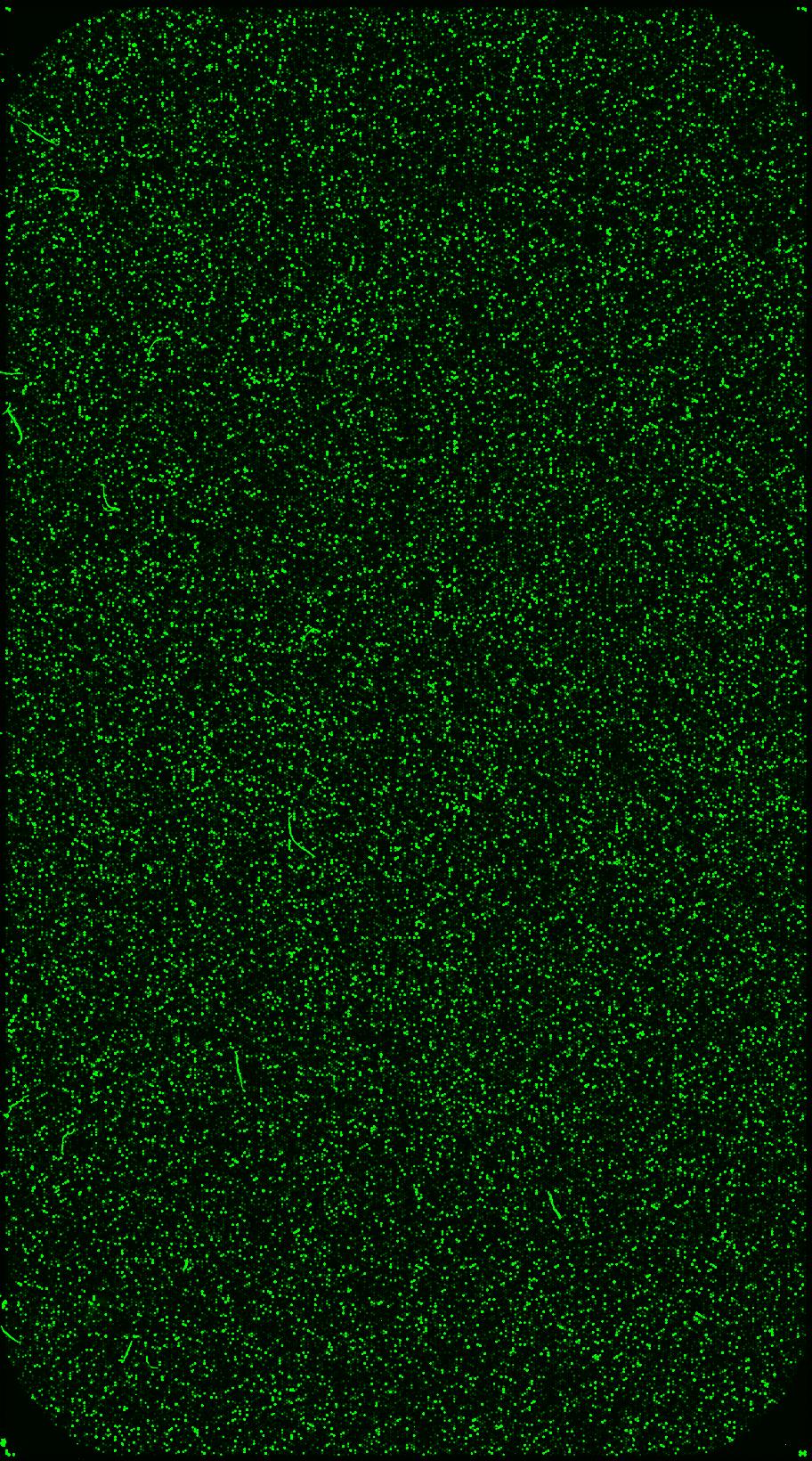


**Figure 3 FaDu - ceRNA Microarray**

**Figure 4 FaDu/DDP4 - ceRNA Microarray**


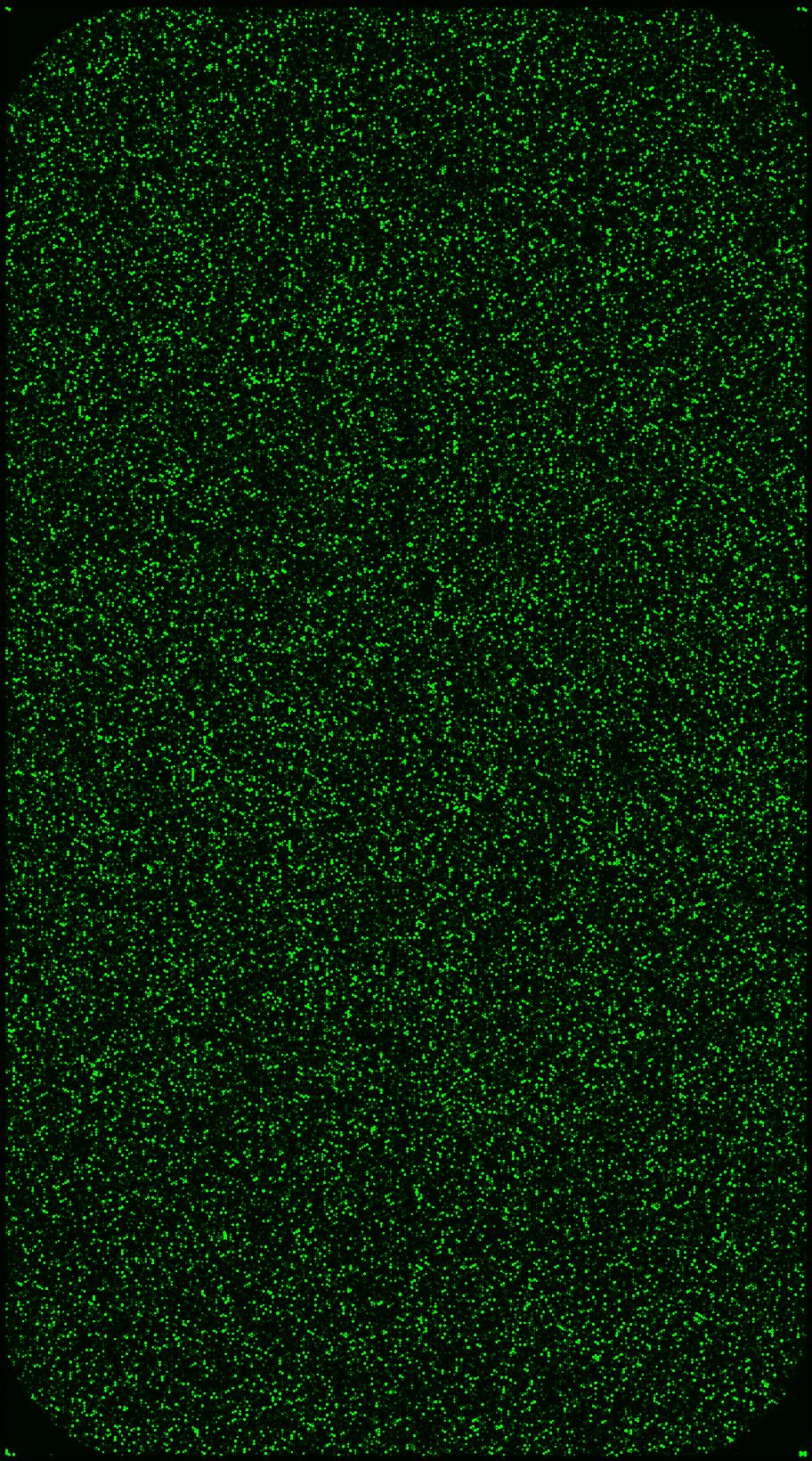

Supplement: Supplemental Information 10 [file peerj-09-11645-s010.docx]
